# Amyotrophic lateral sclerosis-linked FUS/TLS alters stress granule assembly and dynamics

**DOI:** 10.1186/1750-1326-8-30

**Published:** 2013-08-31

**Authors:** Desiree M Baron, Laura J Kaushansky, Catherine L Ward, Reddy Ranjith K Sama, Ru-Ju Chian, Kristin J Boggio, Alexandre J C Quaresma, Jeffrey A Nickerson, Daryl A Bosco

**Affiliations:** 1 Department of Neurology, University of Massachusetts Medical School, Worcester, MA, USA; 2 Department of Cell and Developmental Biology, University of Massachusetts Medical School, Worcester, MA, USA; 3 Department of Biochemistry and Molecular Pharmacology, University of Massachusetts Medical School, Worcester, MA, USA

**Keywords:** Stress granule, Amyotrophic lateral sclerosis, Frontotemporal lobar degeneration, FUS/TLS, Oxidative stress

## Abstract

**Background:**

Amyotrophic lateral sclerosis (ALS)-linked fused in sarcoma/translocated in liposarcoma (FUS/TLS or FUS) is concentrated within cytoplasmic stress granules under conditions of induced stress. Since only the mutants, but not the endogenous wild-type FUS, are associated with stress granules under most of the stress conditions reported to date, the relationship between FUS and stress granules represents a mutant-specific phenotype and thus may be of significance in mutant-induced pathogenesis. While the association of mutant-FUS with stress granules is well established, the effect of the mutant protein on stress granules has not been examined. Here we investigated the effect of mutant-FUS on stress granule formation and dynamics under conditions of oxidative stress.

**Results:**

We found that expression of mutant-FUS delays the assembly of stress granules. However, once stress granules containing mutant-FUS are formed, they are more dynamic, larger and more abundant compared to stress granules lacking FUS. Once stress is removed, stress granules disassemble more rapidly in cells expressing mutant-FUS. These effects directly correlate with the degree of mutant-FUS cytoplasmic localization, which is induced by mutations in the nuclear localization signal of the protein. We also determine that the RGG domains within FUS play a key role in its association to stress granules. While there has been speculation that arginine methylation within these RGG domains modulates the incorporation of FUS into stress granules, our results demonstrate that this post-translational modification is not involved.

**Conclusions:**

Our results indicate that mutant-FUS alters the dynamic properties of stress granules, which is consistent with a gain-of-toxic mechanism for mutant-FUS in stress granule assembly and cellular stress response.

## Background

Mutations in the gene encoding fused in sarcoma/translocated in liposarcoma (FUS/TLS or FUS), also known as the heterogeneous nuclear ribonucleoprotein (hnRNP) P2 [1], are linked to inherited cases of amyotrophic lateral sclerosis (ALS) [2,3]. ALS is a fatal neurodegenerative disease characterized by motor neuron loss, progressive muscle weakening and paralysis [4]. Most ALS-linked FUS mutations are located within the C-terminal nuclear localization signal (NLS) that binds transportin, the nuclear importer that translocates FUS from the cytoplasm into the nucleus [5,6]. Although FUS is predominately localized to the nucleus in most cell types [7], it has nucleo-cytoplasmic shuttling capabilities that may be important for mRNA transport [8]. In fact, FUS is thought to play a role in local translation at the dendrites of neuronal cells [9-11]. Disruption of the FUS/transportin interaction leads to nuclear depletion with concomitant cytoplasmic accumulation of FUS in cultured mammalian cells [6]. The potential relevance of this interaction is underscored by the cytoplasmic accumulation of FUS in both ALS [2,3] and frontotemporal lobar degeneration (FTLD) [12] post-mortem central nervous system (CNS) tissues.

The extent to which FUS mutants redistribute to the cytoplasm correlates with ALS disease severity [6,13]. For example, individuals with the FUS R495X mutation, which leads to truncation of the NLS and significant cytoplasmic retention of FUS, exhibit early disease onset and a relatively severe disease course [13]. Nuclear depletion of FUS may impair putative nuclear functions involving mRNA [14,15] and DNA [16,17] processing. An alternative, though not mutually exclusive, possibility is that mutant-FUS exerts a gain-of-toxic function in the cytoplasm [18].

Recently, a two-hit model has been proposed to account for cytoplasmic FUS toxicity in ALS and FTLD [19]. Cytoplasmic mislocalization of FUS, either through genetic mutations or other unidentified factors, represents the first hit. The first hit alone may not be sufficient to cause disease. However, a second hit stemming from cellular stress directs cytoplasmic FUS into stress granules. Stress granules are stalled translational complexes that form as a normal response to induced stressors such as oxidation, heat-shock, viral infection or hypoxia [20]. The function of stress granules is thought to be in the triage of mRNAs that are destined for expression, storage or degradation, which in turn restores cellular homeostasis [21]. Stress granule function may not be limited to mRNA processing, as the activity of certain proteins can also be controlled by their sequestration into stress granules [22]. It follows that the association of mutant-FUS with stress granules may impair stress response and ultimately cause disease [23]. This notion is supported by evidence of stress granule marker proteins within the pathological aggregates of neurodegenerative disease tissues [6,24,25].

While we and others have firmly established a mutant-specific phenotype with respect to FUS in stress granules [5,6,13,26-30], there is little evidence that mutant-FUS actually alters the properties of stress granules. Although there is no functional assay per se for stress granules, the properties of stress granules that are thought to be relevant to their function include assembly kinetics, dynamics, morphology and abundance [21,31]. Under conditions of oxidative stress, we show that mutant-FUS delays stress granule formation in mammalian cell culture. Once sodium arsenite-induced stress granules are formed, however, those containing mutant-FUS are more dynamic, larger and more abundant compared to stress granules lacking FUS. Upon removal of stress, stress granules disassemble more rapidly in cells expressing cytoplasmic mutant-FUS. Further, we identified the RGG domains within FUS as playing a key role in the assembly of mutant-FUS into stress granules, although the methylation of arginine residues within these RGG domains does not play a significant role. The evidence presented here supports the hypothesis that the association of mutant-FUS with stress granules represents a gain-of-toxic interaction in ALS pathogenesis.

## Results

### The expression of cytoplasmic mutant FUS influences stress granule assembly and disassembly

**Figure 1:**
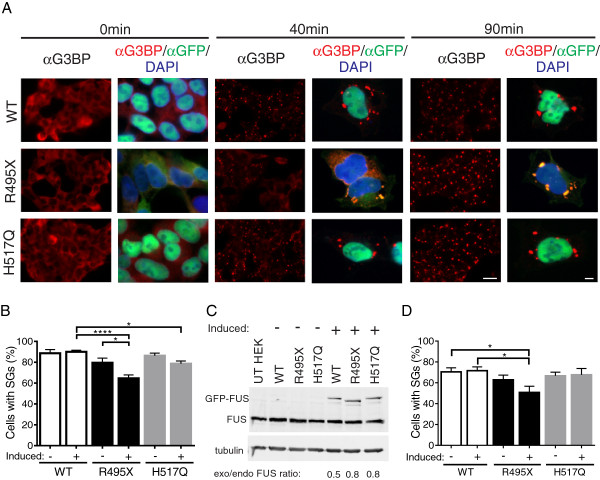
**Mutant-FUS expression delays the assembly and expedites the disassembly of stress granules in human cells. (A)** Representative fluorescence images of HEK-293 cells expressing GFP-FUS (WT, R495X or H517Q) upon treatment with 0.25 mM sodium arsenite for 0, 40 and 90 min. The extent of stress granule formation in each line is illustrated with low magnification (40x) images using the anti-G3BP (red) stress granule marker (columns 1, 3 and 5). The localization of GFP-FUS (green) with respect to the nuclei (DAPI; blue) and stress granules is demonstrated within high magnification (100x) images (columns 2, 4 and 6). Scale bar = 20 and 5 μm, respectively, in low and high magnification images. **(B)** Quantification of the percentage of cells with stress granules at 40 min for the lines in (A) that are either induced (+) or not induced (−) to express GFP-FUS reveal that expression of GFP-FUS R495X causes a significant decrease in the number of cells with stress granules compared to controls. All error bars represent SEMs. Statistically significant differences were determined by one-way ANOVA and Tukey’s post-hoc test (****P < 0.0001, *P < 0.05). Additional significant comparisons include, but are not shown for clarity: uninduced GFP-FUS WT versus induced GFP-FUS R495X (P <0.001), uninduced GFP-FUS H517Q versus induced GFP-FUS R495X (P <0.001), and induced GFP-FUS H517Q versus induced GFP-FUS R495X (P < 0.01). **(C)** Western blot and densitometry analyses of cell lines in **(B)** and naive HEK-293 cells (UT) reveal that exogenous (exo) GFP-FUS proteins are expressed at levels similar to each other and within 2-fold of endogenous (endo) FUS (exo/endo FUS ratio). **(D)** Stress granule disassembly was assessed as described in **(B)** for GFP-FUS WT, R495X and H517Q lines after stress was removed for 90 min. Significantly fewer GFP-FUS R495X expressing cells contain stress granules.

**Figure 2:**
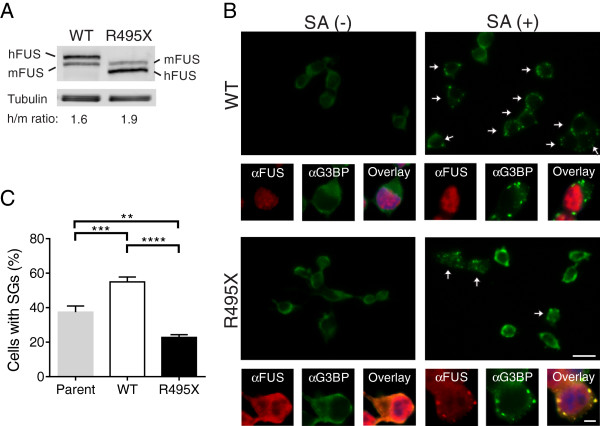
**Expression of the FUS R495X mutant interferes with stress granule assembly in neuronal cells. (A)** Exogenous human (h) FUS proteins are similar to each other and within 2-fold of endogenous mouse (m) FUS as determined by western blot and densitometry analyses. The ratio of hFUS/mFUS proteins is indicated for each cell line. **(B)** Immunofluorescence images of NSC-34 cells expressing untagged FUS (WT and R495X) either in the absence (−) or presence (+) of 0.25 mM sodium arsenite (SA) for 1 hr. Low magnification (40x) images of SA (+) treated cells using the anti-G3BP stress granule marker (green) show a greater number of stress granule-positive cells (denoted by arrows) in the FUS WT line compared to the FUS R495X line. High magnification (100X) images showing a single cell reveal the localization of FUS (red) and G3BP (note: the far red channel was used for detection of G3BP and the images are shown in green for clarity) under all conditions. DAPI (nuclei) is included in the overlay images. Note that in cells with stress granules, cytoplasmic R495X co-localizes to these structures as expected (see overlay image in bottom right). Scale bar = 20 and 5 μm, respectively, in low and high magnification images. **(C)** Quantitative analysis (as described in Figure 1B) of the percentage of NSC-34 cells with stress granules after 1 hr of sodium arsenite treatment revealed that expression of FUS R495X inhibited stress granule assembly compared to cells expressing FUS WT and the parent line. All error bars represent SEMs. Statistically significant differences were determined by one-way ANOVA and Tukey’s post-hoc test (****P < 0.0001, ***P < 0.001, **P < 0.01).

We and others previously demonstrated that ALS-linked FUS mutants assemble into stress granules to an extent that directly correlates with their cytoplasmic mislocalization [6,13]. These experiments were performed under conditions of acute stress such that stress granules assembled rapidly and did not address whether mutant-FUS affects the processes of stress granule assembly and disassembly. First, we employed our previously characterized, doxycycline-inducible HEK-293 cell lines expressing GFP-tagged wild-type FUS and ALS-linked FUS mutants (R495X and H517Q) [13] to examine the effect of mutant-FUS on stress granule assembly under conditions of oxidative stress. The R495X mutation truncates the nuclear localization signal (NLS), causing FUS to significantly redistribute from the nucleus to the cytoplasm (Figure 1A and [13]). On the opposite end of the mislocalization spectrum is H517Q, an autosomal recessive mutation in the NLS that induces a mild mislocalization phenotype (Figure 1A and [2,13]).

Stress granule assembly was initiated in GFP-FUS (WT, R495X, and H517Q) cell lines with 0.25 mM sodium arsenite, an inducer of oxidative stress [32] and an environmental toxicant that can cause neural defects [33,34]. Sodium arsenite has been shown to induce the incorporation of cytoplasmic FUS-mutants into stress granules, but does not influence endogenous FUS or exogenously expressed GFP-FUS WT proteins [13,35]. In fact there is no difference in cellular response to sodium arsenite with respect to stress granule formation or cell viability when FUS expression is reduced [31,35]. Therefore, sodium arsenite induces a mutant-specific phenotype with respect to FUS localization to stress granules. The concentration of sodium arsenite employed here was reduced from the 0.5 mM concentration that is typically used [13,36] in order to lengthen the timescale of stress granule assembly and resolve differences in this process between cell lines. Ras GTPase-activating protein-binding protein 1 (G3BP), a stress granule specific marker [36], was used to probe for stress granules by immunofluorescence (IF) over a 90 min time course of sodium arsenite exposure. At 0 min, there were no stress granules; G3BP remained diffusely cytoplasmic and no cytoplasmic GFP-FUS puncta were detected in any cell line (Figure 1A). By 90 min, all cell lines contained stress granules in virtually every cell. As expected, GFP-FUS R495X co-localized with G3BP in stress granules, as did GFP-FUS H517Q albeit to a far lesser degree. In contrast, GFP-FUS WT remained entirely nuclear and was not detected within these structures (Figure 1A and Additional file 1). Strikingly, fewer GFP-FUS R495X expressing cells contained G3BP-positive stress granules compared to GFP-FUS (WT and H517Q) lines after approximately 40 min of sodium arsenite exposure (Figure 1A).

Quantification of these results revealed that the greatest difference in cells containing stress granules occurred between the GFP-FUS R495X (65 ± 3.3%) and GFP-FUS WT (90 ± 1.5%) lines after 40 min of sodium arsenite exposure (Figure 1B). Stress granules were also assembled in GFP-FUS H517Q cells (79 ± 2.5%), though to a lesser extent than WT cells. Therefore, the expression of mutant-FUS is not sufficient to cause a delay in stress granule assembly, but rather, the delay in assembly depends on the extent that FUS is redistributed to the cytoplasm (WT<H517Q<R495X). Artifacts from FUS over-expression are not expected to influence these results since the expression levels of all exogenous GFP-FUS proteins were similar to each other and within two-fold of endogenous FUS (Figure 1C). To rule out an inherent difference between cell lines irrespective of GFP-FUS expression, identical experiments were performed in uninduced cells (i.e., without doxycycline). Uninduced GFP-FUS (WT and H517Q) lines did not express detectable GFP-FUS and behaved similarly to induced GFP-FUS WT cells, confirming that GFP-FUS WT expression has no effect on stress granule assembly. There was a small yet insignificant difference between uninduced GFP-FUS (WT and R495X) lines (Figure 1B). We suspect this may be due to low levels, below the limit of detection via western analysis (Figure 1C), of GFP-FUS R495X expression in the absence of doxycycline. Nonetheless, induced expression of GFP-FUS R495X in these cells significantly attenuated the assembly of stress granules compared to the uninduced condition in the same line (Figure 1B).

The reversible nature of stress granules is an important functional feature of these structures; upon removal of stress, stress granules disassemble as the cell re-establishes homeostasis. Since mutant-FUS delays the assembly of stress granules, we sought to determine whether mutant FUS also influences the reverse process of disassembly. Cells were treated with 0.25 mM sodium arsenite for 1 h, at which point ~100% of cells in all cell lines contained stress granules. The disassembly process was initiated by replacing sodium arsenite with fresh media, and the percentage of cells with stress granules was quantified after 90 min. At this time point, fewer GFP-FUS R495X expressing cells (51 ± 6.0%) contained stress granules compared to GFP-FUS WT (72 ± 3.7%) and GFP-FUS H517Q (68 ± 6.0%). Therefore, expression of GFP-FUS (WT and H517Q) had virtually no effect, while expression of the cytoplasmic GFP-FUS R495X exerted the most pronounced effect on the kinetics of stress granule disassembly (Figure 1D). Interestingly, a similar effect on stress granule assembly and disassembly was observed upon depletion of endogenous TAR DNA-binding protein 43 (TDP-43) [37], which, unlike FUS, is thought to play a normal role in arsenite-induced stress response [31]. Thus, both a loss of TDP-43 function and a potential toxic gain of mutant-FUS function manifests in delayed stress granule assembly and more rapid stress granule disassembly under conditions of oxidative stress.

While HEK-293 GFP-FUS lines are ideal for microscopic measurements of stress granule properties, owing to the GFP tag on FUS and the relatively flat nature of these cells, we wanted to both confirm these stress granule assembly trends in neuronal cells and rule out a potential “tag effect” from GFP. To this end, motor neuron-like NSC-34 [38] cell lines were engineered to constitutively express untagged human FUS WT or FUS R495X using a lentiviral transduction expression system. FUS protein expression in these cells was accomplished with an IRES-containing bicistronic vector (CSCW2-IRES-GFP), which allowed for the simultaneous expression of both FUS and GFP separately (i.e., not as a fusion protein) but from the same RNA transcript. Therefore, GFP served as a reporter for cells transduced to express either untagged FUS WT or R495X. The expression levels of FUS (WT and R495X) proteins were similar to each other and within two-fold of endogenous FUS (Figure 2A and Additional file 2). In the absence of stress, elevated levels of cytoplasmic FUS were observed in NSC-34 cells expressing FUS R495X, and diffuse cytoplasmic G3BP signal was observed in both FUS R495X and WT cells. Upon exposure to 0.25 mM sodium arsenite for 1 hr, FUS was detected within G3BP-positive stress granules only in cells expressing FUS R495X. Although sodium arsenite exposure lead to fewer NSC-34 cells with stress granules overall compared to HEK-293 cells, the same phenotype was observed in that fewer cells formed stress granules in FUS R495X expressing cells compared to those expressing FUS WT (Figure 2B). Quantification of this phenotype revealed a 2.4-fold lower percentage of stress granule-containing NSC-34 cells for FUS R495X (23 ± 1.6%) compared to FUS WT (55 ± 2.8%) lines (Figure 2C). To assess whether the expression of exogenous human FUS proteins had any effect on stress response in these cells, the percentage of cells with stress granules was also quantified in the parent, or non-transduced, NSC-34 cell line after 1 h of sodium arsenite exposure. While FUS WT and R495X lines exhibited the most significant difference, there were also more FUS WT cells with stress granules relative to the parent (37 ± 3.6%) line (Figure 2C). Possible explanations for why an effect of exogenous FUS WT is observed in NSC-34 but not HEK-293 (Figure 1B) cells is that the expression of exogenous human FUS WT exerts an additional stress due to i) higher relative protein levels (compare exogenous to endogenous FUS in Figures 1C and 2A), and/or ii) different species of cells (expression of human FUS in mouse versus human cells), either of which could heighten the stress response of these cells to sodium arsenite. In fact, expression of FUS WT is sufficient to induce stress and toxicity in different model organisms [39-41]. Nonetheless, the efficiency of stress granule assembly in the parent NSC-34 line is more similar to that of FUS WT than FUS R495X (Figure 2C). Moreover, one would expect FUS R495X to also impose an additional stress, and yet stress granule assembly is attenuated in these cells, providing further evidence that expression of mutant-FUS interferes with the assembly of stress granules under conditions of stress.

Unlike HEK-293 cells (Figure 1), the percentage of NSC-34 cells containing stress granules never reached 100% in any line, and the FUS WT line always contained double the percentage of cells with stress granules compared to FUS R495X. This behavior precluded our ability to perform a similar disassembly analysis as described above for HEK-293 cells (Figure 1D), which compared the absolute percentage of cells with stress granules at different time points.

### The expression of cytoplasmic mutant-FUS alters the dynamic binding properties of stress granule-associated proteins

**Figure 3:**
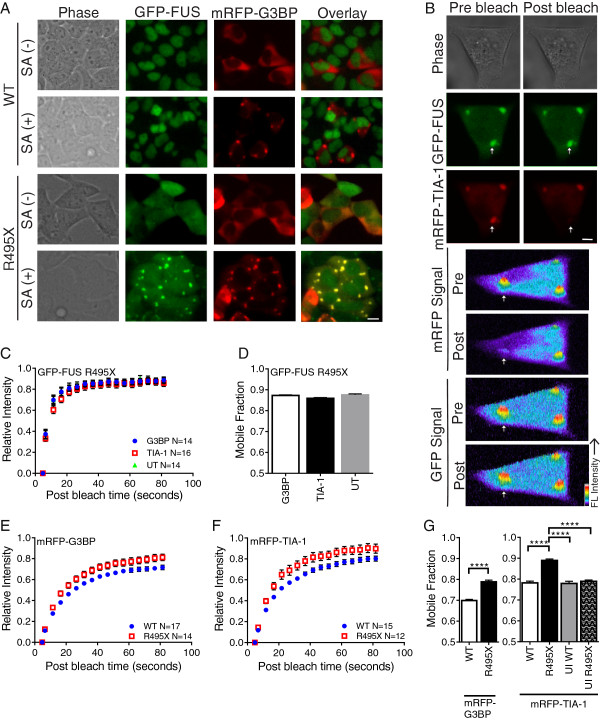
**GFP-FUS R495X is weakly bound to stress granules and alters binding of stress granule-associated proteins. (A)** Live cell images of GFP-FUS (WT and R495X) expressing HEK-293 cells transfected with mRFP-G3BP. Images are shown before (−) and after (+) treatment with 0.2 mM sodium arsenite (SA) for 1 hr. Scale bar = 10 μm. **(B)** Top three panels: exemplar GFP and mRFP images of a SA treated cell for a mRFP-TIA-1 FRAP experiment before and after photobleaching. The mRFP signal, but not GFP signal, is lost from the stress granule (indicated by arrow). Scale bar = 5 μm. Bottom four panels: fluorescence intensity profiles corresponding to the above panels (rotated 90° clockwise). **(C)** The recovery curve for GFP-FUS R495X in untransfected (UT; green triangle) cells are indicative of fast fluorescence recovery. The GFP-FUS R495X profile does not change upon transfection with either mRFP-G3BP (blue circle) or mRFP-TIA-1 (red square). **(D)** Nearly identical mobile fractions support the conclusions in **(C)**. **(E & F)** Recovery curves for mRFP-G3BP **(E)** and mRFP-TIA-1 **(F)** differ for GFP-FUS WT (blue circle) and R495X (red square) expressing cells. **(G)** Mobile fractions for the curves in **(E & F)** are significantly higher for GFP-FUS R495X (black bars) relative to GFP-FUS WT (white bars) cells. Mobile fractions for mRFP-TIA-1 are the same for the following control experiments: GFP-FUS WT expressing cells (white bars), uninduced (UI) GFP-FUS WT cells (grey bar) and uninduced GFP-FUS R495X cells (hatched bar). Asterisks indicate statistically significant differences between cell lines as determined by two-way ANOVA and Tukey’s post-hoc test (****P < 0.0001) on data from at least n=2 independent experiments. All error bars represent SEMs. The total number (N) of stress granule analyzed is indicated on the recovery panels.

Stress granules are highly dynamic structures [32,42,43]. Proteins and mRNA continuously shuttle in and out of stress granules, reversibly binding to other stress granule components in a manner that is thought to regulate both protein signaling activity [22] and mRNA fates towards translational arrest, expression or decay [20]. To study the effect of stress granule-associated mutant-FUS on the dynamic properties of stress granules, we employed fluorescence recovery after photobleaching (FRAP). FRAP measures the relative kinetics and affinities of protein binding within complexes, such as stress granules, over the time course of the experiment [44,45]. Because FRAP reports on binding events in live cells, fluorescently tagged-proteins were employed for these experiments [32,42,43,46]. We transiently expressed monomeric RFP (mRFP)-tagged proteins T-cell-restricted intracellular antigen-1 (TIA-1) or G3BP, two established stress granule-associated proteins, to mark stress granules for FRAP within HEK-293 GFP-FUS (WT and R495X) cells. Overexpression of G3BP is sufficient to form inclusions that resemble stress granules in the absence of stress [31,47]. Since it is not clear whether these G3BP overexpression-induced stress granules have different properties than sodium arsenite-induced stress granules, conditions were optimized for transfection of mRFP-G3BP that minimized the formation of G3BP-positive inclusions *a priori* of sodium arsenite treatment (Figure 3A; see Materials and methods).

During a FRAP experiment on stress granules, the fluorescence from a tagged species (GFP-FUS R495X, mRFP-TIA-1 or mRFP-G3BP in our case) is bleached. The fluorescence signal recovers as bleached molecules unbind from sites in the stress granules, un-bleached fluorescent molecules exchange back into the photobleached area and then bind. The fluorescence recovery time is limited by either diffusion, which is faster than the rates we report here, or by binding kinetics; thus, proteins that are tightly bound to other proteins or cellular structures exhibit relatively long half times of fluorescence recovery (*t*_*1/2*_) [44]. The population of fluorescent molecules that exchange with their bleached counterparts over the time course of the experiment comprise the “mobile fraction” [44]. The “immobile fraction” is the population that is tightly bound and does not exchange over the time course of the experiment. Here, each experiment was carried out such that the photobleaching occurred in only a single channel and fluorescence was diminished evenly over the entire stress granule (Figure 3B). Initially, the dynamics of GFP-FUS R495X within stress granules were investigated since the dynamic binding properties of this protein under stress conditions have not been reported. GFP-FUS R495X cells alone or transfected with either mRFP-G3BP (Figure 3A) or mRFP-TIA-1 (Figure 3B) were exposed to 0.20 mM sodium arsenite for 1 hr, at which point the majority of cells contained fully formed stress granules. The fast recovery (*t*_*1/2*_ of 3.6 ± 2.1s; Figure 3C) of GFP-FUS R495X by FRAP shows that this protein re-binds within stress granules relatively quickly compared to other stress granule-associated proteins (see below). Moreover, ~87% of GFP-FUS R495X molecules constituted the mobile fraction, indicating that GFP-FUS R495X is weakly bound within stress granules (Figure 3D). Neither the *t*_*1/2*_ nor the mobile fraction of GFP-FUS R495X changed significantly upon transient transfection of either mRFP-G3BP or mRFP-TIA-1 (Figure 3C and D). Therefore, neither the process of transient transfection itself, nor the over-expression of stress-granule associated proteins, influenced the dynamic properties of mutant-FUS.

Next we performed FRAP on mRFP-G3BP or mRFP-TIA-1 to determine the effect of mutant-FUS on the dynamic properties of proteins within stress granules. Fluorescence recovery for mRFP-G3BP (*t*_*1/2*_ 12 ± 4.4s; 70 ± 2% mobile) and mRFP-TIA-1 (*t*_*1/2*_ 12 ± 3.9s; 78 ± 2% mobile) within GFP-FUS WT expressing cells was observed (Figure 3E and F). Our measurements of mRFP-TIA-1 in control HEK-293 cells were similar to those reported for GFP-TIA-1 COS7 cells [32]. In cells expressing GFP-FUS R495X, the fluorescence recovery half times were nearly the same for mRFP-G3BP (*t*_*1/2*_ 11 ± 2.8s) and mRFP-TIA-1 (*t*_*1/2*_ 10 ± 2.8s). However, the mobile fraction for both mRFP-G3BP and mRFP-TIA-1 increased significantly in GFP-FUS R495X cells to 79 ± 3% and 89 ± 4%, respectively, compared to GFP-FUS WT cells (Figure 3E-G). Control experiments in GFP-FUS WT (induced and uninduced) and GFP-FUS R495X (uninduced) cells confirmed that an increase in mRFP-TIA-1 mobile fraction required the expression of mutant-FUS (Figure 3G). The increased mobile fraction for mRFP-G3BP and mRFP-TIA-1 indicates that these proteins bind more weakly to factors within GFP-FUS R495X positive stress granules compared to stress granules lacking mutant-FUS. As a result, there is increased exchange of mRFP-G3BP and mRFP-TIA-1 between the area that is photobleached and the area that is not photobleached, resulting in fluorescence recovery. Together, these data demonstrate that the incorporation of mutant-FUS into sodium arsenite-induced stress granules decreases the binding of other stress granule-associated proteins within these structures.

Because we and others observed that stress granules form as a result of G3BP overexpression [31,47], we examined whether their dynamic properties were different compared to those stress granules induced by sodium arsenite. mRFP-G3BP exhibited significantly weaker binding (i.e., larger mobile fraction, P < 0.05) within stress granules in GFP-FUS WT cells under conditions of G3BP over-expression compared to sodium arsenite stress, indicating that the dynamic properties of these stress granules are inherently different (Additional file 3). The most striking difference was the effect of mutant-FUS: GFP-FUS R495X significantly *increased* mRFP-G3BP binding within stress granules (i.e., smaller mobile fraction) under conditions of G3BP over-expression compared to all other conditions, which is the opposite trend in sodium arsenite-induced stress granules (Figure 3). Therefore, the effect of GFP-FUS R495X on the dynamic properties of stress granules depends on the stressor and is consistent with observations that the source of stress influences the constituents within stress granules [48]. Irrespective of the stress, our results show that the incorporation of GFP-FUS R495X into stress granules alters the dynamic binding interactions of other well-characterized stress granule-associated proteins within in these structures.

### Expression of mutant-FUS increases the size and abundance of stress granules

**Figure 4:**
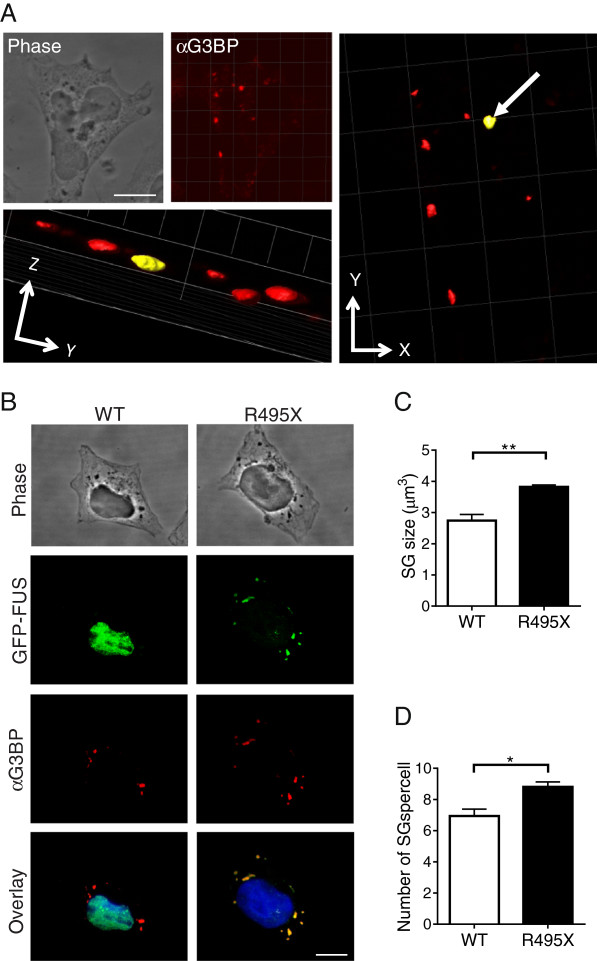
**Stress granule size and number are increased in GFP-FUS R495X expressing cells. (A)** Clockwise order: representative single-plane phase (top left) and anti-G3BP (stress granule marker; red) immunofluorescence (top middle) confocal images of an HEK-293 cell expressing GFP-FUS WT treated with 0.5 mM sodium arsenite for 1 hr. Three-dimensional (3D) reconstruction was used to quantify stress granule volume (see Materials and methods, and C within this figure). The *xy* (right) and *yz* (bottom; view along arrow in xy image) planes for this 3D reconstructed image are shown; the stress granule highlighted in yellow is marked for volume analysis. Scale bar = 10 μm. **(B)** Representative maximum projection confocal images of GFP-FUS WT and GFP-FUS R495X expressing cells treated as in **(A)** exemplify the size and number of stress granules for each line. Scale bar = 10 μm. **(C and D)** GFP-FUS R495X expressing cells contain stress granules with larger volume **(C)** and in greater abundance **(D)** relative to cells expressing GFP-FUS WT. Asterisks indicate statistically significant differences between cell lines as determined by the Student’s t-test (**P < 0.01, *P < 0.05) on data from n=3 independent experiments. All error bars represent SEMs.

In addition to dynamics, stress granule size and abundance could also be altered by the presence of mutant-FUS. As such, both stress granule size and abundance were quantified in HEK-293 cell lines expressing either GFP-FUS (WT or R495X) after exposure to 0.5 mM sodium arsenite for 1 hr, at which point stress granules were fully formed (>95% of cells contain stress granules). In contrast to previous methods that measure the area of stress granules, which does not take into account the three-dimensional aspect of these structures, we developed a method to quantify the volume of stress granules based on fluorescence intensity measurements (see Materials and methods). Briefly, fixed cells were labeled with an anti-G3BP antibody and then optically sectioned by confocal microscopy. Three-dimensional reconstruction of these sections allowed us to quantify the volume of selected stress granules (Figure 4A). This analysis revealed that GFP-FUS R495X expressing cells produced significantly larger stress granules (3.8 ± 0.1 μm^3^) compared to GFP-FUS WT cells (2.7 ± 0.2 μm^3^; Figure 4B and C). This trend is consistent with ALS-linked TDP-43 mutants, which also show an increased stress granule size under conditions of hyperosmolar stress [49], suggesting that this may be a common disease-related characteristic.

Our quantitative analysis also revealed that mutant-FUS expressing cells contained 27% more stress granules per cell (8.8 ± 0.3) compared to WT-FUS (6.9 ± 0.4; Figure 4B and D). This result is consistent with a recent report demonstrating a greater abundance of stress granules within patient FUS (R521C and R514G) fibroblast lines under conditions of sodium arsenite relative to control lines [30]. Moreover, cells expressing ALS-linked TDP-43 also produce more stress granules compared to control cells under stress [25]. Therefore, disease-causing FUS and TDP-43 proteins appear to alter stress granules morphology by increasing their size and abundance.

### The RGG domains play a key role in modulating the association of mutant-FUS with stress granules

**Figure 5:**
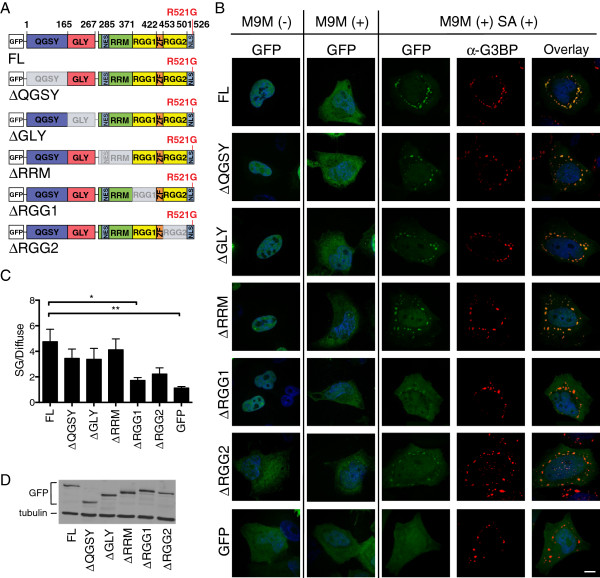
**The RGG domains modulate the incorporation of FUS into arsenite-induced stress granules. (A)** Illustration of full length (FL) GFP-FUS R521G and constructs lacking the following sequences: Gln-Gly-Ser-Tyr-rich (∆QGSY), Gly-rich (∆GLY), RNA recognition motif (∆RRM), and Arg-Gly-Gly-rich (RGG) regions (∆RGG1 and ∆RGG2). **(B)** Confocal images of HeLa cells transfected with GFP-FUS R521G constructs (green) alone (−) or co-transfected (+) with MBP-M9M, the transportin-1 inhibitor. Note the increased levels of cytoplasmic GFP-FUS in co-transfected cells (compare columns 1 and 2). Confocal fluorescence images of co-transfected cells treated with 0.5 mM sodium arsenite for 1 hr were used to assess the ability of GFP-FUS R521G constructs (green; column 3) to associate with stress granules (G3BP; red; column 4). The greatest degree of GFP and G3BP co-localization was observed in cells expressing FL GFP-FUS R521G, whereas there was minimal co-localization in control cells expressing free GFP (compare panels in column 5). **(C)** Quantitative analysis (see Materials and methods) of **(B)** reveals that constructs lacking RGG domains (∆RGG1 and ∆RGG2) exhibit impaired localization to stress granules. Statistically significant comparisons include FL and ∆RGG1 (*P < 0.05), FL and GFP (**P < 0.01), and ∆QGSY and GFP (*P < 0.05; not shown on graph for clarity) by one-way ANOVA followed by a Dunnett’s post-hoc test on n=3 independent experiments. All error bars represent SEMs. **(D)** Western blot analysis of HeLa cells in **(B)** demonstrates equivalent expression levels for all GFP-FUS R521G constructs.

In order to understand the factors modulating the incorporation of mutant FUS into stress granules, we engineered GFP-tagged FUS constructs lacking the functional domains of FUS and tested their ability to incorporate into stress granules. The functional domains of FUS are as follows: the prion-like QGSY-rich region (QGSY), a glycine-rich region (GLY), an RNA recognition motif (RRM), and two arginine-glycine-glycine-rich domains (RGG1 and RGG2) separated by a C2-C2 zinc finger motif (ZF) (Figure 5A). All of these domains have been shown to play a role in modulating the incorporation of other RNA binding proteins into stress granules [26,50-52] and thus each have the potential to influence the association of mutant-FUS with stress granules. The extent with which FUS mutants localize to arsenite-induced SGs correlates with their level of cytoplasmic expression (Figures 1, 2 and [13]). However, since transient transfection of highly cytoplasmic FUS mutants (e.g., R495X) has the potential to produce cytoplasmic protein aggregates that could confound our stress granule analysis [13], domain deletion constructs were engineered within the background of the less aggregation-prone R521G mutant.

Transient transfection of GFP-FUS R521G deletion constructs in HeLa cells resulted in varying degrees of GFP-FUS cytoplasmic expression: FL, ∆QGSY, ∆RGG1, ∆GLY and ∆RRM were predominately expressed in the nucleus, whereas relatively high cytoplasmic expression was observed for ∆RGG2, which lacks part of the signal used for nuclear import of FUS [5] (Figure 5B). Deletion of the zinc finger motif resulted in a construct that failed to express in mammalian cells (data not shown), possibly because this construct is unstable and/or structurally altered [53]. Although GFP-FUS R521G exhibits cytoplasmic expression upon transient transfection [2], the levels here were not sufficient for an accurate stress granule analysis. It was therefore necessary to increase the cytoplasmic expression of FUS-deletion constructs by co-transfection with maltose binding protein (MBP)-tagged M9M. M9M is a transportin/Kapβ2-specific nuclear import inhibitor that blocks the nuclear import of FUS [6,54]. Co-transfection with MBP-M9M increased cytoplasmic retention of GFP-FUS for all constructs, ensuring that each construct had equal potential to assemble into stress granules (Figure 5B).

Administration of 0.5 mM sodium arsenite for 1 hr induced stress granule formation (Figure 5B) in ~75% of cells for all constructs. A ratio of the GFP signal within stress granules relative to the diffuse GFP-FUS signal in the cytoplasm (stress granule/diffuse FUS signal) was used to quantify the incorporation of each construct within stress granules. Full length GFP-FUS R521G exhibited a robust localization to stress granules with a stress granule/diffuse FUS ratio of 4.75 +/− 0.97 (Figure 5B and C). As expected for a protein that does not associate with stress granules, analysis with free GFP as a negative control produced the lowest ratio of 1.13 +/− 0.11. GFP-FUS R521G ΔQGSY, ΔGLY, and ∆RRM were not significantly different from FL GFP-FUS R521G. In contrast, deletion of the RGG1 domain (ΔRGG1) significantly impaired the localization of FUS into stress granules (1.72 +/− 0.22), and deletion of the RGG2 domain (∆RGG2) exhibited a similar impairment trend (2.23 +/− 0.48). We note that while distinct FUS-positive stress granules are observed for ∆RGG2, a comparatively high level of GFP-FUS signal remains diffuse, yielding a low stress granule/diffuse FUS ratio. All constructs were expressed at comparable levels (Figure 5D) and a threshold was applied (see Materials and methods) such that all cells included in this analysis expressed similar levels of FUS. Together, these data demonstrate that the RGG domains in FUS are the most important for mutant-FUS localization to stress granules.

### Methylation of mutant-FUS is not required for its assembly into stress

**Figure 6:**
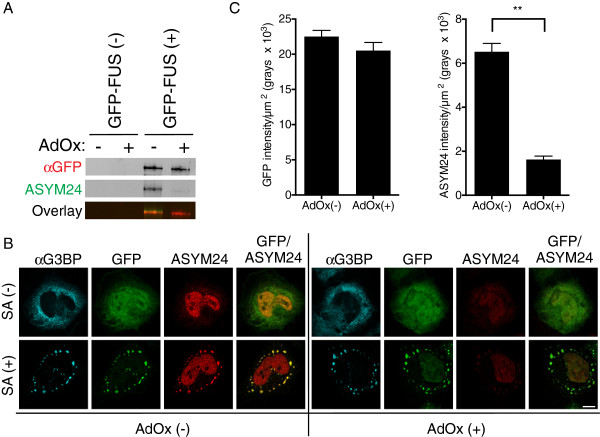
**Methylation of mutant-FUS is not required for its assembly into stress granules.** HeLa cells transfected with GFP-FUS R495X were pre-treated with the methyltransferase inhibitor adenosine-2,3 dialdehyde (AdOx) and then exposed to sodium arsenite to promote stress granule assembly. **(A)** GFP-FUS R495X was detected by western analysis only for immunoprecipitation (IP) reactions from transfected cells (GFP-FUS (+)). Anti-GFP was used for both IP and western analyses. The ASYM24 antibody revealed that GFP-FUS R495X was hypomethylated on arginine residues when cells were pre-treated with AdOx. **(B)** Confocal fluorescence images revealed a robust association of GFP-FUS R459X with sodium arsenite-induced (SA(+)) stress granules, both in the absence (left panels) and presence (right panels) of AdOx. Conversely, the ASYM24 signal within the same stress granule was dramatically attenuated when cells were pre-treated with AdOx. **(C)** Quantification of n=3 independent experiments from **(B)** further supports the conclusions above. Statistical significance was determined by a Student’s t-test (** P < 0.01). All error bars represent SEMs.

In light of the 20 dimethylated arginine residues within the RGG domains of FUS [55], and the fact that arginine dimethylation dictates protein and RNA interactions as well as protein subcellular localization [56], we explored the possibility that arginine dimethylation of mutant-FUS regulates its association with stress granules. While methylated FUS has been detected within stress granules in cell culture and pathological CNS inclusions from individuals harboring FUS mutations [5], it is not clear whether methylation is actually required for FUS incorporation into these structures. To investigate this question, HeLa cells were pre-treated with the global methyltransferase inhibitor adenosine 2,3-dialdehyde (AdOx) [57] prior to expression of the highly cytoplasmic GFP-FUS R495X mutant and sodium arsenite exposure. We note that conditions (see Materials and methods) were used to minimize GFP-FUS R495X aggregation in these experiments. Immunoprecipitation of GFP-FUS R495X using an anti-GFP antibody, followed by western analysis with the dimethyl-specific ASYM24 antibody, confirmed that GFP-FUS R495X is hypomethylated in the presence of AdOx (Figure 6A). Despite the significant degree of hypomethylation, GFP-FUS R495X maintains a robust association with stress granules under these conditions (Figure 6B and C). Conversely, the overall ASYM24 signal, including the signal within stress granules, is significantly attenuated. Similar results were seen in our stable HEK-293 GFP-FUS R495X line (data not shown). While the small population of methylated GFP-FUS R495X that remains after AdOx treatment (Figure 6A) could be sequestered into stress granules, the significant decrease in ASYM24 signal (i.e., the decrease in signal for methylated proteins) with no decrease in GFP-FUS signal argues against this possibility. Therefore, while methylated mutant-FUS can assemble into stress granules, this post-translational modification is not a prerequisite for its incorporation.

## Discussion

The association of cytoplasmically mislocalized ALS-linked FUS mutants with stress granules is well established [5,6,13,26-30], but what affect does mutant-FUS have on the properties of stress granules? We sought to examine the effect of mutant-FUS on the physical properties of stress granules that are potentially linked to function. While there is no functional assay per se for stress granules, they are believed to represent sites of mRNA triage, which influences whether particular mRNA transcripts are retained within stress granules, translated on ribosomes or degraded within P-bodies [20]. There is also evidence that the signaling activity of proteins can be controlled by their sequestration and/or release from stress granules during stress [22]. Thus, the cellular response to stress is modulated, at least in part, by stress granules. Since mutant-FUS, but not WT-FUS, is incorporated into stress granules under various induced stressors, the mutant protein has the potential to disturb stress granules and impair cellular stress response in ways that could contribute to ALS pathogenesis [19].

Stress granules assemble in response to induced stress. Our results show that ALS-linked, cytoplasmic FUS R495X delays the assembly of stress granules in both HEK-293 (Figure 1) and neuronal NSC-34 (Figure 2) cells under conditions of acute oxidative stress. The predominantly nuclear FUS H517Q mutant also delays stress granule assembly, but to a lesser degree than FUS R495X (Figure 1). Therefore, the delay in stress granule assembly correlates with cytoplasmic levels of mutant-FUS, probably because the protein is poised to enter stress granules once stress is induced. Since over-expression of some ALS-FUS mutants reportedly induce the spontaneous formation of cytoplasmic inclusions that stain positively for stress granule markers [28,29], one might expect the expression of mutant-FUS to correlate with a faster rate of stress granule assembly. However, the properties of stress granules are influenced by the nature of the induced stressor [48], and we show that stress granules induced by protein over-expression exhibit different dynamic properties than those induced by sodium arsenite (Additional file 3). We also demonstrate that mutant-FUS accelerates the disassembly of stress granules (Figure 1D). Therefore, expression of mutant-FUS appears to disfavor the formation of and/or destabilizes stress granules, possibly by interfering with protein interactions within these structures (Figure 3). The effects of mutant-FUS on stress granule assembly and disassembly are reminiscent of effects seen during TDP-43 knock-down [37]. Considering that stress granule assembly is a regulated process [20], factors that either delay or accelerate stress granule assembly/disassembly may adversely affect cellular homeostasis.

Interestingly, once stress granules are formed, mutant-FUS exerts an effect on both stress granule morphology and abundance that may appear counterintuitive based on the effect of FUS during the processes of assembly and disassembly. While the expression of GFP-FUS R495X both disfavors stress granule assembly and weakens stress granule associated interactions, under conditions of persistent stress the size and abundance of stress granules is augmented by the expression of mutant-FUS (Figure 4). This increased size and abundance of stress granules does not necessarily mean these structures are held together more tightly, but rather is a likely consequence of the additional protein load associated with these structures from the GFP-FUS R495X protein itself. This rationale may also be relevant to the increased size of stress granules in ALS-linked TDP-43 mutants under conditions of hyperosmolar stress [49] and suggests that this phenotype may be part of a common disease pathway. An intriguing, but not mutually exclusive, possibility is that mutant-FUS and TDP-43 recruit additional protein partners and mRNA substrates into stress granules, thereby further increasing their size and abundance (Figure 4 and [25,30]). Indeed, thousands of mRNA transcripts are bound by FUS [58,59] with many distinct mRNAs bound by cytoplasmic mutant-FUS but not WT FUS [59]. Therefore, mutant-FUS may inappropriately process mRNAs and/or facilitate aberrant cytoplasmic protein interactions during stress. The latter possibility is supported by our FRAP analyses, which showed that both mRFP-G3BP and mRFP-TIA-1 exhibit weaker binding and heightened dynamics within sodium arsenite-induced stress granules containing mutant-FUS (Figure 3). In fact, GFP-FUS R495X altered the dynamic properties of stress granules in all of our FRAP experiments, raising the possibility that mutant-FUS interferes with the sorting mechanisms [21,22] associated with these structures under stress.

If the association of mutant-FUS with stress granules does indeed represent a gain of toxic interaction, it will be important to identify factors that modulate this association. Although FUS contains multiple domains that contribute to FUS aggregation [60] and/or are homologous to sequences that direct other proteins into stress granules [26,50-52], our results show that the RGG domains are largely responsible for directing FUS into stress granules (Figure 5). Our results are in general agreement with a recent report by Bentmann et al., which also demonstrated a key role for the RGG domains in assembling FUS into stress granules [26]. However, our results do not support a role for the Gly-rich and RRM domains in this process, whereas the former study did. This discrepancy may be due to the difference in FUS constructs, stressor (sodium arsenite versus heat shock [26]) and/or the FUS mutation (R521G versus P525L [26]) that were employed in these studies. Whether the RNA-binding ability of FUS is required for its localization to stress granules is not altogether clear [26,27]. Several domains within FUS exhibit RNA-binding capabilities, including the RMM, RGG, and zinc finger domains. Bentmann et al. demonstrated a correlation between cytoplasmic FUS constructs that bound RNA and were incorporated into stress granules, consistent with a role for RNA-binding in the assembly of mutant-FUS into stress granules [26].

That the RGG domains direct mutant-FUS to stress granules raises the possibly that this process is controlled by arginine dimethylation of RGG motifs [61]. Emerging evidence indicates that the RGG motifs within FUS are methylated by protein arginine N-methyltransferase-1 (PRMT1) [55,62,63], and that this post-translational modification can influence the subcellular localization of mutant-FUS [62,63]. While stress granules contain methylated proteins (Figure 6B), and methylated forms of mutant-FUS have been detected in both stress granules and diseased-tissues [5], our data suggests that methylation of FUS is not a prerequisite for its incorporation into stress granules (Figure 6).

How might the incorporation of mutant-FUS into stress granules alter cellular homeostasis under conditions of induced stress, and what are the implications for neurodegenerative disease? We show that mutant-FUS delays stress granule assembly (Figures 1 and 2), decreases the binding of stress granule-associated proteins within stress granules (Figure 3), and increases both size and abundance of stress granules (Figure 4). These physical and dynamic properties of stress granules are thought to be linked to stress granule function, and thus the effects of mutant-FUS may culminate in impaired stress response and, eventually, in neurodegeneration. Although there have been no reports of overt cytotoxicity in mutant-FUS cellular models exposed to sodium arsenite or other stressors, the effects of impaired stress response may appear more distinctly as a function of age, disease progression and/or chronic stress in the human disease [19]. In fact, stress granule marker proteins have been detected within the pathological inclusions of CNS tissues from patients with ALS and FTLD [6,25], supporting the notion that stress response factors are altered during the course of disease. Moreover, these observations raise the possibility that stress granules are precursors to the end-stage aggregates that are characteristic of these diseases [23]. Although our data show that mutant-FUS accelerates stress granule disassembly, under conditions of persistent stress these granules containing mutant-FUS are larger and more numerous and thus have the potential to coalesce into larger aggregates. Extending analyses of stress granules to other models systems, such as human iPS cells from individuals with ALS or ALS rodent models, may allow us to better address whether altered stress granule assembly plays a role in disease onset and/or progression, and whether the association of ALS-linked proteins with stress granules does in fact impact disease.

## Conclusions

ALS-linked FUS mutants that mislocalize to the cytoplasm not only incorporate into stress granules under conditions of oxidative stress, but the presence of mutant-FUS in stress granules alters the properties of these structures. Expression of mutant-FUS delays the assembly and expedites the disassembly of stress granules. Furthermore, the morphology and dynamics of these structures is influenced by the presence of mutant-FUS. Therefore, our data are consistent with a gain of toxic function for mutant-FUS with respect to stress granule assembly and dynamics.

## Materials and methods

### Cell culture and drug treatments

Inducible GFP-FUS expressing FlpIn HEK-293 cells were maintained as described previously [13]. Human cervical carcinoma cells (HeLa) were maintained in Modified Eagle’s medium (MEM, Gibco 10370) supplemented with 10% (v/v) heat inactivated fetal bovine serum (Sigma, F4135), 2 mM L-glutamine (Gibco, 25030), and 1% (v/v) penicillin and streptomycin solution (Gibco, 15140). Mouse motor neuron–like hybrid cell lines (NSC-34) [38] constitutively expressing untagged human FUS were maintained in Dulbecco’s modified Eagle’s medium (Invitrogen, 11965118) supplemented with 10% (v/v) tetracycline-tested fetal bovine serum (Sigma, F6178), and 2 μg/mL puromycin (Invitrogen, A11138-03).

NSC-34 cells constitutively expressing untagged human FUS constructs were generated by lentiviral transduction of the CSCW2-IRES-GFP lentivector (a generous gift from Dr. Miguel Esteves, University of Massachusetts Medical School) containing FUS (WT or mutant R495X). Flow cytometry was used to enrich for expression of the GFP reporter in each line. Cells with equivalent levels of exogenous FUS proteins were employed.

For drug treatments, the following stocks were prepared and stored at freezing temperatures: 50 mg/mL doxycycline (Sigma, D9891) in water (−80°C), 100 mM sodium arsenite (Sigma, 71287) in water (−20°C) and 20 mM adenosine-2,3 dialdehyde (“AdOx”; Sigma, A7154) in water (−20°C). FUS expression in the FlpIn HEK-293 lines was induced with the addition of 1 μg/mL doxycycline for 24 hrs unless otherwise noted. Cells were then exposed to sodium arsenite and/or AdOx as described below.

### Plasmids and cloning

The pEmRFP-G3BP and mRFP-TIA-1 plasmids for FRAP analyses were generously provided by Drs. Nancy Kedersha and Paul Anderson (Brigham and Women’s Hospital, Harvard Medical School). The mRFP-TIA-1 was sub-cloned into the low expression lentivirus vector CShPW2 (a gift from Miguel Estevez, UMMS) with NheI and Knp1 restriction sites using BP Clonase II (Invitrogen, 11789–020), thereby creating CShPW2-RFP-TIA-1. MBP-M9M was a kind gift from Dr. Yuh Min Chook (University of Texas Southwestern Medical Center).

GFP-FUS R521G deletion constructs were constructed as follows: PCR amplified full length GFP-FUS R521G, flanked by attB homologous sequences, was cloned into pDONR221 vector (Invitrogen, 12536–017) with BP Clonase II (Invitrogen, 11789–020) to generate the starting plasmid pDONR221:GFP-FUS R521G. To facilitate substitution of the full length gene with deletion/truncation variants, restriction sites for KpnI and XbaI were introduced upstream of the ATG start codon and downstream the TAA stop codon, respectively, using the following primers: **fwd**: GGGGACAAGTTTGTACAAAAAAGCAGGCTGGTACC**ATG**GCCTCAAACGATTATACCC, **rev**: GGGGACCACTTTGTACAAGAAAGCTGGGTTCTAGA**TTA**ATACGGCCTCTCCCTGC. To generate deletion constructs, the following primers were designed by joining the upstream and downstream sequences flanking the domain that was deleted: ∆**GLY_fwd**: AGAACCAGTACAACAGCAGCAGTACCATCTTTGTGCAAGGCC; ∆**GLY_rev**: ACTCAATTGTAACATTCTCACCCAGACTGCCAGACAACAACACCCGGGCAGACTTTAATCGGG; ∆**RRM_rev**: CCACGACCATTGCCACCACCGTTGTTGTCTGAATTATCCTGTTCG; ∆**RGG1_fwd**: CAAGGTCTCATTTGCTACTCGCGCTGGTGACTGGAAGTGTCC; ∆**RGG1_rev**: CATATTCTCACAGGTGGGATTAGGCCGATTAAAGTCTGCCCGGC; ∆**RGG2_fwd**: CCAGTGTAAGGCCCCTAAACCAGATAAGATGGATTCCAGGGGTGAGCAC; ∆**RGG2_rev**: GTGCTCACCCCTGGAATCCATCTTATCTGGTTTAGGGGCCTTACACTGG; ∆**422-526_fwd**: GGGGACAAGTTTGTACAAAAAAGCAGGCTGGTACC**ATG**GCCTCAAACGATTATACCC; ∆**422-526_rev**: GGGGACCACTTTGTACAAGAAAGCTGGGTTCTAGA**TTA**TCGCTGCTGTCCTCCACC. The deletion reactions were performed using the pDONR221:FUS R521G plasmid as template and the QuikChange II Mutagenesis kit (Stratagene; 200523) according to the manufacturer’s instructions. For the ∆QGSY truncation construct, PCR was performed using a reverse primer for the full-length R521G gene paired with a forward primer containing the 5’-end sequences of ∆GLY flanked by the restriction enzyme KpnI recognition sequence: ∆**QGSY_fwd**: CAGGCTGGTACCGGTGGTGGAGGTGGAGGT. All constructs were then sub-cloned into the expression vector pDEST-53 (Invitrogen) using Gateway cloning method with LR Clonase II (Invitrogen, 11791–100) according to the manufacturer’s instructions.

### Immunofluorescence

Standard immunofluorescence protocols were employed as described previously [13]. Briefly, cells were fixed with 4% paraformaldehyde for 10–15 min then blocked with PBSAT (1X PBS/1% BSA/0.5% Triton-X 100) for 30–60 min at ambient temperature. Primary antibodies described in each experiment were diluted in PBSAT and applied to cells at ambient temperature for 1 hr. Primary antibody dilutions were as follows: 1:2000 for mouse anti-G3BP (BD Transduction Labs, 611126), 1:1000 for rabbit anti-G3BP (Proteintech, 130-57-2AP), 1:1500 for rabbit anti-dimethyl arginine (“ASYM24”; Millipore, 07–414) and 1:200 mouse anti-FUS (Santa Cruz, SC-4771). Cells were then incubated with secondary antibodies diluted 1:1000–1:2500 in PBSAT for 45 min at ambient temperature. Secondary antibodies included Dylight 549 conjugated anti-mouse IgG (Jackson ImmunoResearch Labs, 715-505-151), Cy3 conjugated anti-mouse IgG (Jackson ImmunoResearch Labs, 715-165-151), Cy3 conjugated anti-rabbit IgG (Jackson ImmunoResearch Labs, 711-165-152), and Cy5 conjugated anti-mouse IgG (Jackson ImmunoResearch Labs, 715-175-151). GFP signal was enhanced by 1:1000 dilution of Alexa Fluor 488-conjugated rabbit anti-GFP (Invitrogen, A21311). Cells were stained with 34 ng/mL DAPI in dH_2_O, and coverslips were mounted with ProLong Gold anti-fade reagent (Invitrogen, P36930).

### Western blotting

Standard western blotting protocols were employed as described previously [13]. Primary antibodies described in each experiment were diluted as follows: 1:1000 for mouse anti-GFP (Living Colors; Clontech, 632380), 1:1000 for rabbit anti-dimethyl-arginine (“ASYM24”; Millipore, 07–414), 1:1000 for mouse anti-tubulin (Sigma, T9026), and 1:1000 for rabbit anti-FUS. Rabbit anti-FUS antibodies were generated by GenScript against a C-terminal epitope, using the peptide CKFGGPRDQGSRHDSEQDNSD. Blots were incubated with primary antibodies for 1 hr at ambient temperature or overnight at 4°C. Secondary antibodies, including anti-mouse IRDye 680 (Licor, 926–32220) or IRDye 800 (LiCor, 926–32210) and anti-rabbit IRDye 680 (LiCor, 926–32220) or IRDye 800 (Licor, 926–32211), were diluted 1:10000 and incubated with blots for 1–2 hrs at ambient temperature. Bands were visualized with an Odyssey Infrared Imager (LiCor, Model 9120), and densitometry measurements performed with the Odyssey Software (LiCor, V3.0).

### Stress granule assembly and disassembly kinetics

Inducible GFP-FUS (WT, R495X and H517Q) HEK-293 cells were plated on coverslips at a density of 5 × 10^4^ cells/coverslip. The next day, GFP-FUS expression was induced as described above. For stress granule assembly measurements, cells were treated with 0.25 mM sodium arsenite for 40 or 90 min. Coverslips were then fixed and processed for immunofluorescence (IF) with the mouse anti-G3BP and rabbit anti-GFP antibodies listed above. For stress granule disassembly measurements, cells were treated with 0.25 mM sodium arsenite for 60 min, at which time the media containing sodium arsenite was replaced with fresh media. After 90 min in fresh media, cells were processed for IF as described above. The percentage of cells with stress granules was determined as [(the number of cells containing at least one stress granule / total number of cells) × 100]. More than 2000 healthy, interphase, non-crowded cells were counted in multiple (between n=4 and n=11, depending on cell line and condition) independent experiments for both assembly and disassembly conditions. A one-way ANOVA with Tukey's multiple comparisons post-test was used to compare the induced and uninduced groups. Similar parameters were used for assembly kinetics in NSC-34 parent cells and cells expressing untagged human FUS proteins with the following changes: cells were treated with sodium arsenite for 1 hr, and immunofluorescence was performed with the rabbit anti-G3BP and mouse anti-FUS antibodies listed above. More than 2000 cells were counted in at least n=11 independent experiments per condition. Statistical significance was determined by one-way ANOVA with Tukey's multiple comparisons post-test.

### Fluorescence recovery after photobleaching

GFP-FUS WT and GFP-FUS R495X HEK-293 cells were plated at a density of 8 ×10^4^ cells/plate in 35 mm glass bottom dishes (MatTek Corp, P35GC-1.5-14-C), allowed to adhere for 48 hrs, then transfected with either CShPW2-RFP-TIA-1 or pEmRFP-G3BP expression plasmids using Lipofectamine 2000 (Invitrogen, 11668–019) according to the manufacturer’s instructions with a 1.6 μl Lipo: 3.2 μg DNA ratio. Approximately 23 hr post-transfection, phenol red(−) growth media without (overexpression experiments) or containing 0.2 mM sodium arsenite (stress granule experiments) was applied to the cells for 1 hr prior to FRAP.

FRAP experiments were performed at 37°C as previously described [45]. Multiple cells were analyzed in each experiment over a 30 min period starting at 60 min of sodium arsenite exposure. Experiments were carried out on a Leica SP5 AOBS laser scanning confocal microscope using a 40× 1.3NA water immersion objective or a Leica SP1 system using a 40×, 1.25NA oil immersion objective. No more than two stress granules, from opposite sides of a cell, were individually bleached using a 1-3s laser pulse delivered by a 488 nm or 561 nm laser. A pre-bleach, immediate post-bleach and 16 additional post-bleach images spaced at 5 sec intervals were captured. Leica Confocal Software (Leica Microsystems, Exton, PA) was used to measure fluorescence intensity in the bleached region of interest (ROI), the whole cell, and in a background control area lacking cells at each time point. The data was analyzed and background fluorescence subtracted using Excel. The relative fluorescence intensity (I_rel_) in the bleached area was calculated as previously shown [45]. Briefly, the following equation was used: I_rel, t_ = (I_t_ × (C_0_/C_t_))-(I_pbl_ × (C_0_/C_bl_))/(I_0_-(I_pbl_ × (C_0_/C_pbl_)), where C_0_ is the total cellular fluorescence before bleaching, C_pbl_ is the total cellular fluorescence in the post-bleach image, C_t_ is the total cellular fluorescence at time t, I_0_ is the pre-bleach ROI fluorescence intensity, I_t_ is the ROI fluorescence intensity at time t, and I_pbl_ is the post-bleach ROI fluorescence intensity. The data was normalized using this equation such that the post-bleach ROI fluorescence intensity was set to 0 and the pre-bleach ROI fluorescence intensity to 1. I_t_ was calculated as the percentage difference between the relative fluorescence asymptote of the recovery curve and a relative recovery of 1, a value that would reflect complete recovery without an immobile fraction. Recovery curves were drawn using Graphpad Prism 6 (Graphpad Software), with individual time points presented as means ± SEMs. Fluorescence recovery half times were calculated from exponential one-phase association curves best fit for the recovery graphs: F(t) = F_max_ (1- e^-kt^). Mobile fractions were calculated from the plateau region from each curve, which was identified as the series of data points with < 2% change in slope over time.

At least two independent experiments were performed for all conditions. The total number of stress granules analyzed for each condition is shown in Figure 3. A two-way ANOVA was used to determine statistical significance between the mobile fractions for each of the experiments.

### Morphology experiments

Stable GFP-FUS HEK-293 cells were plated and processed for immunofluorescence as described under ‘stress granule assembly and disassembly kinetics’ above, except that cells were treated with 0.5 mM sodium arsenite for 1 hr. Confocal stacked images (0.2 μm stack step, 4 μm range) were acquired using a Zeiss Axiovert 200 microscope with a PerkinElmer UltraView LAS spinning disc equipped with a 100× phase objective. Imaris analytical software (Bitplane Scientific Software) was used to construct 3D projections of image stacks. Volume measurements were taken of each stress granule with a G3BP fluorescence signal that was at least 2-fold above background. Because P-bodies had an average volume of 0.5 μm^3^ (data not shown) by the same analysis, only stress granules with a volume > 0.5 μm^3^ were included in the analysis. Data is averaged from three independent experiments per line, using approximately 30 stress granules per condition. Statistical significance was determined using the Student’s t-test.

### Analysis of FUS deletion constructs in stress granule assembly

HeLa cells were plated on coverslips at a density of 2.5 × 10^4^ cells/coverslip and adhered at 37˚C for 24 hrs, after which GFP-FUS R521G truncation constructs and MBP-M9M were transiently transfected into cells using Lipofectamine-2000 (Invitrogen, 11668) according to the manufacturer’s instructions. Twenty-four hours post transfection, cells were subjected to media containing 0.5 mM sodium arsenite for 1 hr. Cells were processed immunofluorescence with mouse anti-G3BP as described above.

Confocal microscopy was performed using a Solamere Technology Group CSU10B spinning disk confocal system equipped with a Yokogawa CSU10 spinning disk confocal scan head. Image stacks (0.2 μm stack step; 13 stack range) were acquired using a 100× oil objective, a Roper Cool-snap HQ2 camera and MetaMorph V7.6.3 software. Background signal was subtracted by removing fluorescence from a dark-current image (acquired with the laser off) from each raw image. For the GFP images, variations in illumination and detection efficiencies at each pixel were corrected by dividing the dark-adjusted intensities by a normalized flat-field image of a uniformly green fluorescent slide (Chroma Technology, Rockingham, VT, USA) acquired using the same 525/50 nm band-pass filter. Four channels were imaged per cell: FITC for GFP-FUS, Cy3 for G3BP, DAPI for nuclei and phase for cell borders.

The extent of GFP-FUS incorporation into stress granules was analyzed with MetaMorph V7.6.3 software using the Integrated Morphometry Analysis tool. Since cells with a GFP signal brighter than 1.5 × 10^6^ grays/μm^2^ tended to form cytoplasmic aggregates *a priori* of arsenite treatment (data not shown), only transfected cells with GFP-FUS expression levels < 1.5 × 10^6^ grays/μm^2^ were selected for analysis. Stress granules were selected using the Cy3 (G3BP) channel as a reference. The slice corresponding to the center of the stress granule was selected from each image stack. Stress granules with an area of at least 0.5 μm^2^ were selected. An outline was drawn around each G3BP granule, and this outline was then transferred to the corresponding FITC (GFP-FUS) image, such that the GFP-FUS signal intensity (“stress granules intensity”) within that granule could be measured. GFP signal intensity measurements were also acquired in the region proximal to the stress granule, and was referred to as the “Diffuse intensity”. The ratio of stress granule intensity (i.e., GFP-FUS inside the stress granule) to diffuse intensity (i.e., GFP-FUS outside the stress granule) was determined for a total of 75–150 stress granules per construct over three independent experiments. Statistical significance was determined by a one-way ANOVA followed by a Dunnett’s post hoc test in Graphpad Prism 6 (Graphpad software).

### Methyltransferase inhibition studies

HeLa cells were plated at a density of 2.5 × 10^4^ cells/coverslip and adhered for 24 hrs, after which GFP-FUS R495X expression constructs were transiently transfected with Lipofectamine-2000 either with or without 25 μM AdOx for 24 hr. Cells were then exposed to 0.5 mM sodium arsenite for 1 hr, and coverslips were processed for immunofluorescence using the mouse anti-G3BP rabbit and ASYM24 antibodies as described above. For quantification, confocal images of 30 cells per condition across three independent experiments were taken, and the intensity of GFP-FUS and ASYM24 signal within stress granules was determined between AdOx-treated and untreated conditions using MetaMorph as described above. Statistical significance was determined using a Student’s t-test.

For GFP immunoprecipitation (IP) reactions, cells were lysed for 15 min in IP buffer (400 μL 1% NP-40 (MP Biomedicals, 198596)/50 mM Tris–HCl (Sigma T3253-500G)/5 mM EDTA (Fisher E478-500)/150 mM NaCl and 10% v/v glycerol (Acros 15982–0010) in water; pH 7.5), and centrifuged at 13000 rpm for 15 min at 4°C. The supernatant was pre-cleared with 50 μL Biomag Protein G beads (Qiagen, 311812) for 2 hrs at 4°C. Anti-GFP-coated beads for each sample were prepared by incubating 0.5 μL of anti-GFP (Abcam, ab290) in 400 μL of IP buffer with 50 μL Biomag Protein G beads for 2 hrs at 4°C. IP reactions were performed at 4°C overnight with 100 μg of pre-cleared lysate. Protein elution was accomplished with 50 μL 1X SDS loading buffer (Boston Bioproducts BP11R) for 5 min at 95°C, and 20 μL of sample was subjected to western blot analysis with mouse anti-GFP, mouse anti-tubulin and ASYM24 as described above.

## Competing interests

The authors declare that they have no competing interests.

## Authors’ contributions

DB, LK, and CW planned and performed the majority of experiments; DB, AJQ and JAN planned, performed and analyzed data for FRAP; RJC cloned deletion constructs for structure-function analyses; RRKS and KB contributed to the design and data interpretation for experiments; DB, LK and DAB wrote the paper. All authors read and approved the final manuscript.

## Supplementary Material

Additional file 1 **A minor fraction of GFP-FUS H517Q incorporates into stress granules in response to sodium arsenite.** Images for the indicated GFP-FUS cell line (top 3 rows) were collected as described in Figure 1A. Antibody markers used for immunofluorescence are indicated on the left. Images overexposed for GFP detection (bottom row) reveal that a minor fraction of GFP-FUS H517Q (green) co-localizes with G3BP-positive stress granules (red). FUS H517Q containing stress granules are denoted by arrows. Conversely, GFP-FUS WT is not detected in stress granule (i.e., there is no GFP-positive signal that co-localized with G3BP), even with high exposure. Scale bar = 5 μm. Click here for file

Additional file 2 **NSC-34 cells expressing untagged human FUS WT and R495X exhibit similar transduction efficiencies.** Fluorescent images of the GFP reporter (green) in NSC-34 cells transduced with lentivirus containing untagged human FUS WT or R495X. Transduction efficiencies of approximately 100% were determined for both lines. Cellular nuclei are stained with DAPI (blue). Scale bar = 20 μm. Click here for file

Additional file 3 **Sodium arsenite induced stressed granules display different dynamics compared to those induced by mRFP-G3BP over-expression. (A)** Transfection of mRFP-G3BP was sufficient to induce G3BP positive stress granules in a subset of both GFP-FUS WT and GFP-FUS R495X cells as determined by live cell imaging. Scale bar = 20 μm. **(B)** The FRAP recovery curve for mRFP-G3BP inside the stress granule in (A) was different depending on whether mRFP-G3BP was transfected into GFP-FUS WT (blue circle) or R495X (red square) expressing cells. Note the trend is opposite from sodium arsenite-induced stress granule in Figure 3. **(C)** Quantification of the mobile fraction from the recovery curves in (B) compared to those in Figure 3G revealed that expression of GFP-FUS R495X significantly increased mRFP-G3BP binding (i.e., smaller mobile fraction) to stress granules in the over-expression condition compared to all other conditions. Asterisks indicate statistically significant differences between cell lines as determined by two-way ANOVA (****P < 0.0001) on data from n=3 independent experiments. Additional significant comparisons include, but are not shown for clarity: WT in the overexpression versus WT in the sodium arsenite condition (P < 0.05); R495X in the overexpression versus R495X in the sodium arsenite condition (P < 0.0001); R495X in the overexpression versus WT in the sodium arsenite condition (P < 0.05). The total number (N) of stress granules analyzed is indicated. All error bars represent SEMs. Click here for file
