# Supplementary figures and images for: Amyotrophic lateral sclerosis-linked FUS/TLS alters stress granule assembly and dynamics
Source: Mol Neurodegener. 2013 Aug 31;8:30. doi: 10.1186/1750-1326-8-30 (PMC3766239; doi:10.1186/1750-1326-8-30)

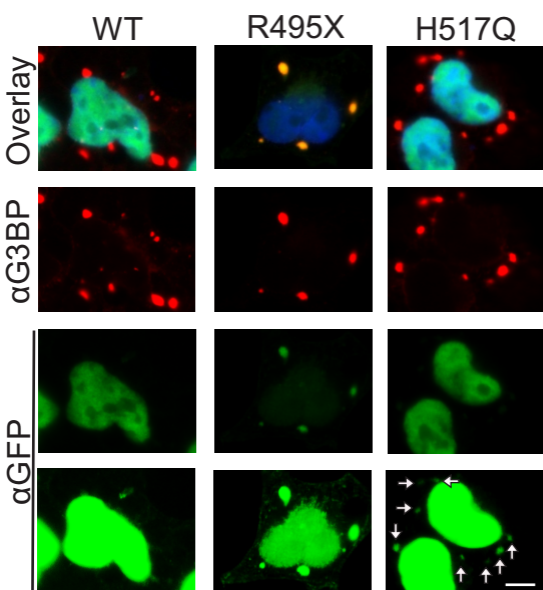

Supp Figure 1

Supplement: Additional file 1 — A minor fraction of GFP-FUS H517Q incorporates into stress granules in response to sodium arsenite. Images for the indicated GFP-FUS cell line (top 3 rows) were collected as described in Figure 1A. Antibody markers used for immunofluorescence are indicated on the left. Images overexposed for GFP detection (bottom row) reveal that a minor fraction of GFP-FUS H517Q (green) co-localizes with G3BP-positive stress granules (red). FUS H517Q containing stress granules are denoted by arrows. Conversely, GFP-FUS WT is not detected in stress granule (i.e., there is no GFP-positive signal that co-localized with G3BP), even with high exposure. Scale bar = 5 μm. [file 1750-1326-8-30-S1.pdf]

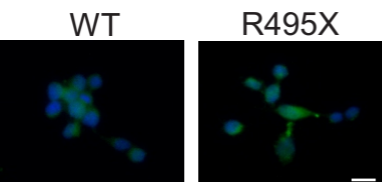

Supp Figure 2

Supplement: Additional file 2 — NSC-34 cells expressing untagged human FUS WT and R495X exhibit similar transduction efficiencies. Fluorescent images of the GFP reporter (green) in NSC-34 cells transduced with lentivirus containing untagged human FUS WT or R495X. Transduction efficiencies of approximately 100% were determined for both lines. Cellular nuclei are stained with DAPI (blue). Scale bar = 20 μm. [file 1750-1326-8-30-S2.pdf]

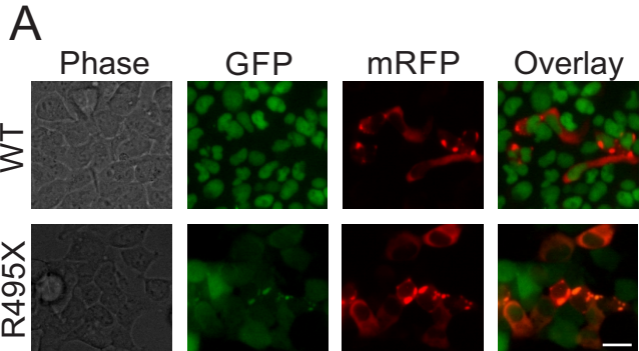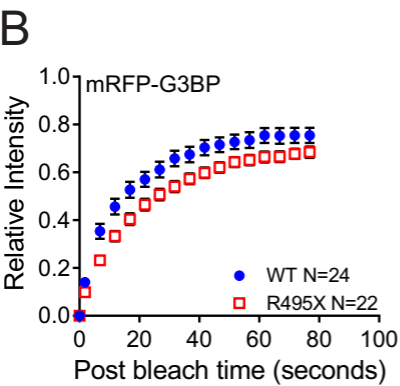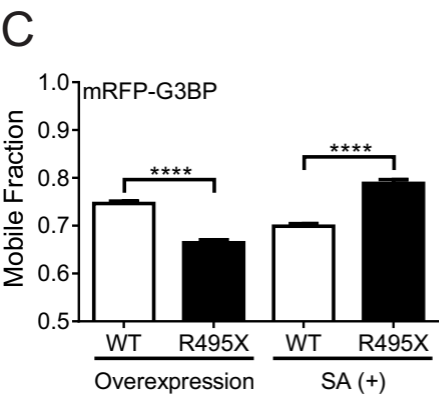

Supp Figure 3

Supplement: Additional file 3 — Sodium arsenite induced stressed granules display different dynamics compared to those induced by mRFP-G3BP over-expression. (A) Transfection of mRFP-G3BP was sufficient to induce G3BP positive stress granules in a subset of both GFP-FUS WT and GFP-FUS R495X cells as determined by live cell imaging. Scale bar = 20 μm. (B) The FRAP recovery curve for mRFP-G3BP inside the stress granule in (A) was different depending on whether mRFP-G3BP was transfected into GFP-FUS WT (blue circle) or R495X (red square) expressing cells. Note the trend is opposite from sodium arsenite-induced stress granule in Figure 3. (C) Quantification of the mobile fraction from the recovery curves in (B) compared to those in Figure 3G revealed that expression of GFP-FUS R495X significantly increased mRFP-G3BP binding (i.e., smaller mobile fraction) to stress granules in the over-expression condition compared to all other conditions. Asterisks indicate statistically significant differences between cell lines as determined by two-way ANOVA (****P < 0.0001) on data from n=3 independent experiments. Additional significant comparisons include, but are not shown for clarity: WT in the overexpression versus WT in the sodium arsenite condition (P < 0.05); R495X in the overexpression versus R495X in the sodium arsenite condition (P < 0.0001); R495X in the overexpression versus WT in the sodium arsenite condition (P < 0.05). The total number (N) of stress granules analyzed is indicated. All error bars represent SEMs. [file 1750-1326-8-30-S3.pdf]
